# The Impact of COVID-19 on Neuropsychological and Emotional-Behavioural Development in a Group of 8- and 9-Year-Old Children

**DOI:** 10.3390/jcm13164768

**Published:** 2024-08-14

**Authors:** Angelica Marfoli, Giulia Speziale, Gaia Del Prete-Ferrucci, Harlan Cole, Angelica De Sandi, Denise Mellace, Daniela Chieffo, Sergio Barbieri, Alberto Priori, Bernardo Dell’Osso, Gabriella Pravettoni, Roberta Ferrucci

**Affiliations:** 1 Department of Oncology and Hemato-Oncology, University of Milan, 20122 Milan, Italygiulia.speziale1996@gmail.com (G.S.);; 2 University of Bath, Bath BA2 7AY, UKharlancole@hotmail.co.uk (H.C.); 3 Foundation IRCCS Ca’ Granda Ospedale Maggiore Policlinico, 20122 Milan, Italy; 4 Department Woman Children and Public Health, Catholic University of the Sacred Heart, 00168 Rome, Italy; 5 Clinical Psychology Unit, Fondazione Policlinico Universitario A, Gemelli, 00168 Rome, Italy; 6 Department of Health Science, “Aldo Ravelli” Center for Neurotechnology and Experimental Brain Therapeutics, University of Milan, 20142 Milan, Italy; 7 Neurology Unit, ASST Santi Paolo e Carlo, 20142 Milan, Italy; 8 Department of Biomedical and Clinical Sciences, University of Milan, 20157 Milan, Italy; 9 Applied Research Division for Cognitive and Psychological Sciences, IEO, European Institute of Oncology IRCCS, 20141 Milan, Italy

**Keywords:** children, COVID-19, development, emotional-behavioural, neuropsychological

## Abstract

**Introduction**: The rapid spread of the COVID-19 pandemic has had a significant impact on the psychological well-being of millions of people around the world, and even more so among children. Contracting SARS-CoV-2, resulting in home confinement and restrictions on daily and school activities, led to negative effects on the mental health of the paediatric population. Although children suffering from COVID-19 had milder general symptoms compared to adults, impairments in cognitive, neuropsychological, and emotional-behavioural development were noted. **Objective**: The main aim of the present study was to detect possible changes in the neuropsychological and emotional-behavioural development of children after infection with the SARS-CoV-2 virus. The second aim was to investigate possible relationships between cognitive abilities and psychosocial characteristics. **Methods**: A total of 40 patients aged 8–9 years were recruited and divided into two groups: children who contracted (CG) and did not contract (NCG) SARS-CoV-2. The BVN 5–11 (Neuropsychological evaluation battery for developmental age from 5 to 11 years) instrument was administered to assess attention, memory, verbal recall, planning, phonemics, and categorical fluency domains in the paediatric population. Data on changes in emotional-behavioural profile and daily activities were collected through a questionnaire to parents. **Results**: The Wilcoxon signed-rank test revealed a significant change in mood after the COVID-19 period only in the CG participants (*p* = 0.019). However, the neuropsychological performance of the two identified groups on BVN 5–11 sub-items was below the cutoff of clinical significance. Correlations were found between sub-items of the BVN 5–11 battery, extracurricular activities, and children’s psycho-motor development. Significant positive correlations were observed between Naming on visual presentation and Reading time (*p* = 0.006), backward digit span and time of motor activity (*p* = 0.009), Visual attention and Reading time (*p* = 0.048), and Phonemic fluency and time observed using devices (*p* = 0.030). Positive statistically significant correlations were also found between Mood and Free behaviour (*p* = 0.000), between Mood and Structured behaviour (*p* = 0.005), and between Mood and peer Interaction (*p* = 0.013). **Conclusions**: SARS-CoV-2 infection negatively affected the emotional development of children contracting the virus. The neuropsychological functioning of the paediatric population was influenced by psychosocial variables and time spent on daily activities, which played a protective role in children’s cognitive development.

## 1. Introduction

Since the beginning of the global COVID-19 pandemic, people around the world have experienced huge changes in their daily routines. Italy was the first European country to experience the unpredictable consequences of the coronavirus disease, with the impact of pandemic containment measures such as school closures and quarantine compromising physical, psychological, and emotional well-being [1,2]. Social distancing, closure of educational services, personal hygiene measures such as hand washing, physical and social isolation, daily routine disruption, financial stress involving high and rising levels of unemployment, and numerous other potential triggers for stress response have all been intensified due to the pandemic, setting up a situation of psychological discomfort, including an overall exacerbation of anxiety and depression symptoms [3,4,5]. Moreover, in addition to the physical symptoms experienced during the SARS-CoV-2 acute phase of the infection, such as fever, cough, shortness of breath, muscle pain, and headache [6], some patients who recovered from COVID-19 experienced a condition called “Long COVID” syndrome. This condition was characterised by lasting physical pain, including symptoms such as persistent myalgia or arthralgia, chest pain, stomach pain, diarrhoea, heart palpitations, and skin lesions [7,8]. There are also behavioural alterations, such as anxiety and depression disorders and sleep disturbance, as well as neurological symptoms, including headache, difficulty in concentrating, and cognitive impairment [9,10,11].

Children represented one of the population sections who experienced the most drastic changes due to the COVID-19 pandemic [12]. Specifically, school closures, social isolation, involving strongly limited social contacts and cancelled out-of-home leisure time activities, routine disruption, interruption of education and nutrition care, reduced physical activities, and excessive screen time contributed to produce sleep dysregulation associated with feelings of uncertainty, distress and anxiety, and depressive symptoms [11]. In fact, even if children who were infected by SARS-CoV-2 exhibited milder symptoms compared to adults [13], it should be noted that COVID-19 may have had an impact on their cognitive functions and psychological well-being. Attention difficulties and concentration problems have been reported by different authors [4,9,10,14] as well as memory impairments [11,14]. In particular, several studies indicated that an increase in distraction and difficulty with schoolwork characterised children’s cognitive development after the COVID-19 period. Higher levels of stress, anxiety, and depression also seemed to compromise their psychological well-being [15,16], with emotional and behavioural distresses such as distraction, irritability, and fear of infection being found [15,17,18].

A Canadian study indicated that children’s risk of poorer mental health due to the COVID-19 pandemic may have depended on risk and protective factors heading into the pandemic. In particular, the results revealed that children had poorer mental health on days when they experienced a COVID-19 stressor (i.e., virtual academic difficulties, social isolation), and that this association was stronger for those with higher pre-pandemic peer victimisation [19]. Further research [1] examined the consequences of this phenomenon, facing difficulties in determining the outcome of potentially distressing situations due to the interaction of various factors, such as the psychological reaction of parents and the correlated children’s response, the family’s lifestyle and well-being before the pandemic, the parent–children relationship before the event, and the ability to cope with adversities in terms of resilience.

## 2. Psychosocial and Cognitive Effects of the COVID-19 Pandemic

COVID-19 produced mild physical symptoms and a low mortality rate in the paediatric population. However, the pandemic situation and the following restrictive measures to limit infections, like school closures, the stopping of recreational activities, the cancellation of events, and limitations to socialisation had severe consequences on children’s psychosocial and cognitive development.

In the following theoretical section, the major psychological, emotional-behavioural, and neuropsychological effects that impacted children’s development and their functioning in adulthood are reported according to the literature.

### 2.1. Psychological, Emotional, and Behavioural Disruption

Literature reports different psychological and emotional-behavioural effects in primary school-age children due to the state of emergency during the COVID-19 pandemic.

A huge percentage of parents (85%) reported the perceived impact of the pandemic on their children’s emotional state and behaviours. Globally, the daily moods of children were more frequently reported as negative, and they felt more insecure, fearful, and isolated [5,6]. There was also an increase in boredom (52%), irritability (39%), feelings of loneliness (31%), restlessness (38%), nervousness (38%), anxiety (28%), anger (25%), reluctance (24%), sadness (23%), and feeling uneasy and worried (30%). The fear of COVID-19, fear of death, fear of relatives’ death, fear of being isolated, and fear of asking questions about the pandemic emerged in greater numbers than in the pre-pandemic period [16,18,20,21,22]. During quarantine, children were more likely to argue with the rest of the family (29.7%), were more dependent on them (28%), and were more affectionate, and separation problems increased [4,5,6]. For example, Orgilés et al. [21] found that Italian children are sadder and lonelier compared to Spanish ones, perhaps due to the longer duration of the Italian quarantine. In fact, during the COVID-19 period, all children reported a higher level of stress, but the ones that experienced quarantine reported statistically significantly more severe fear, nervousness, and annoyance. Specifically, they presented elevated levels of isolation, boredom, and sadness [23]. Moreover, a significant increase in externalising symptoms among children and adolescents was verified, with a consequent 17% meeting the criteria for a long-term diagnosis of externalising disorders and persistent mental health disorders [24]. In a Scottish study, more than half of the parents completing the questionnaire reported that their 8–11-year-old children scored close to the average for conduct problems and emotional symptoms, highlighting the negative impact of the pandemic on the quality of their child’s peer relationships, prosocial behaviour, and social interactions [15].

#### Anxiety and Depression

Recent research has widely confirmed the impact of COVID-19 on the substantial increase in anxiety and depression clinical cases [6,9], with the worldwide prevalence ranging greatly from 17.6% to 43.7% among countries and regions, in line with the proportion of stress varying from 7% to 25% [25].

Several studies revealed that the revolution of daily routines, social distancing and isolation, the rapid growth of infected cases and deaths, and high levels of parental stress created a feeling of uncertainty and anxiety in the paediatric population [15], with an increase of distress and anxiety symptomatology among children aged 6–12 years from 6.5% before the pandemic era to 16/35% [5,14,20,21,22,26,27,28]. This was especially true for quarantined children, who reported a level of stress four times higher than the general paediatric population [17]. However, conflicting results were found by Bignardi and colleagues [29], with small and not statistically significant changes in levels of anxiety.

### 2.2. Extracurricular Activities

Context influenced global well-being, and even if the COVID-19 pandemic and consequent closure and restrictions were not controllable, there was a range of individual actions. Everyone was able to decide the activities to perform at home to maintain healthy lifestyle behaviours, such as correct alimentation, being active, and regular sleep, and to preserve mental and physical health during the lockdown [30].

#### 2.2.1. Physical and Outdoor Activities

Physical Activity (PA) was associated with mood and physical, social, and academic well-being during the COVID-19 pandemic [31]. With the quarantine leading to a change of habits in the entire population, the importance of movement during the forced rest period proved to be very relevant [2]. Some studies have demonstrated the positive impact of home-based physical activity as a good preventive coping strategy to mitigate the consequences of quarantine [32,33]. The WHO recommends at least 60 min of physical activity daily for children between 5 and 17 years old: before the quarantine, 54% of Italian children achieved this mark, while during the quarantine this percentage decreased to 15% [21]. Appropriate movement was positively associated with reduced risk of depression, anxiety, insomnia, tiredness, and loneliness. Additionally, more prosocial and fewer hyperactivity-inattention behaviours were reported in children performing the correct amount of PA [30]. Although 25% of children exercised more compared to the pre-pandemic period, globally, long-term physical inactivity and less outside time during the outbreak were reported [27,34,35,36]. More than half of 8- to 11-year-old girls and boys recalled that confinement had a negative impact on their movement and caused exercise intolerance [14,15,30,37]. Some studies demonstrated that during the early months of the lockdown, children’s physical activity levels decreased, with increased risk for compromised development of the gross motor skills involved in activities like running, jumping, and balancing [24]. Access to outdoor spaces (fairly large gardens/balconies/patios) had a positive impact on families, with the preservation of outdoor activities increasing PA times and alleviating children’s depressive symptom rates [28].

#### 2.2.2. Screen Time

During the COVID-19 outbreak, with the consequent social isolation and school closures, increased sedentary behaviours and screen use (e.g., watching television, using devices, and playing video games) were observed. Their functions were the compensation of education, communication, socialisation, and other aspects of daily life [24,35,38]. A total of 80% of children 8 to 11 years old spent more time on screens than before, both for leisure and social media use [21,36,38,39].

Recent research found no association between screen use during the pandemic and either child executive function or prosocial behaviour decrease, while greater screen time was positively associated with poor performance in the study, decreased real-life social interaction, neglecting personal life, and relationship disorders [40]. In particular, children using electronic devices more than 2–4 h/day seemed to present a higher risk of loneliness as well as depressive and anxiety symptom occurrence. Moreover, electronic media use at night caused adverse consequences in sleep/decreased sleep quality [15,25,41]. Overall, the majority of girls and boys respectively reported spending more time using a cell phone and playing video games during the lockdown, with these sedentary activities representing a way for them to interact with peers (internet gaming, video-call), play with parents, increase cognitive stimulation, and promote healthier emotional well-being.

### 2.3. Cognitive Effects of COVID-19 Pandemic

Cognitive effects in the paediatric population during and after the COVID-19 outbreak were also observed; however, neuropsychological alterations in the paediatric literature are not studied as much as emotional and behavioural ones. There are small findings, the majority obtained by parent questionnaires, where caregivers’ perception of children’s ability modification was reported, while direct data collected from children’s performance is limited [9,10,11]. The most affected domains were memory, attention, concentration, processing speed, and executive functioning, including semantic and phonemic fluency [7].

To better investigate these aspects, the present study was conducted with the aim of detecting any differences between children who had been affected by COVID-19 and those who had not been affected, assuming a greater impairment in the psycho-social, emotional, and neuropsychological development of the first group.

## 3. Methods and Materials

The primary aim of the research project was to screen the neuropsychological skills in 8- and 9-year-old children after the COVID-19 pandemic to detect the presence of modifications, also considering the influence of distance learning. The analysed neuropsychological domains were selective visual attention, short- and long-term memory, immediate verbal recall, planning, phonemics, and categorical fluency. Data were collected from the whole sample, from children who were positive for SARS-CoV-2 (named CG: COVID-19 group) and from children who never experienced this infection (named NCG: non-COVID-19 group).

The second aim of the study was to investigate the possible correlations between children’s cognitive abilities and psychosocial characteristics.

### 3.1. Participant Recruitment and Ethics

This study was conducted among children and parents in Northern Italy, in Valtellina and Valchiavenna areas (Sondrio and Chiavenna provinces) between 8 October and 27 November 2022. The children were subjected to a series of cognitive tests inserted within the playful framework of the “*Cervelliadi*, the brain Olympic game”. The parents answered a paper-and-pen survey. The self-selecting nature of recruitment means that this is not a nationally representative sample. Non-probability sampling (purposive sampling) techniques were used to enrol the participants.

Recruitment took place through two main routes: (1) word of mouth in scholastic and extracurricular contexts (sports, educational) by parents, educators, and children who knew the project; (2) collaboration with public and private centres that made available the physical space in which the research was carried out and contributed to making the project known. Once the parents became aware of the *Cervelliadi* project, they contacted the experimenter by telephone. The telephone interview allowed the parents to obtain more accurate information on the research project. The researcher could conduct an initial screening, making sure that the inclusion and exclusion criteria were respected, as well as defining the logistical details for the execution of the research protocol.

A total of 43 children and their parents completed the test and survey. After cleaning up the sample by excluding ineligible children because they did not meet the specified age range, 40 participants were drawn for the final analysis. Participants’ sociodemographic data are shown in Table 1. The study participants constituted 47.5% males [19] and 52.5% [21] females. The 40 recruited children were non-equally divided between the COVID-19 group (CG), with 29 children, and the non-COVID-19 group (NCG), with 11 children.

Inclusion criteria included children from 7 years and 7 months to 9 years and 6 months, according to the BVN’s (Neuropsychological evaluation battery for developmental age from 5 to 11 years) battery age individuation [42]. The current study did not include children aged less than 7 years and 7 months and older than 9 years and 6 months. Furthermore, children that before the COVID-19 pandemic presented cognitive difficulties and disabilities were excluded in order to obtain generalisable results for the global paediatric population. Before enrolment, parents/caregivers completed and signed the privacy consent and the parents’ informed consent. Children were asked directly if they agreed to participate in the study. In the case of a positive response, they signed an informed consent form for minors. It was also necessary that they attended the full test, completing the entire child registration protocol. The non-completion and/or non-subscription by the parent/caregivers and the minors of the informed consent and the privacy consent constituted criteria for exclusion from the research.

Participants were given no economic motivation. At any time, participants could retire from the study without giving any justification. The study was carried out in accordance with the Helsinki Declaration and good clinical practice regulations after approval from the Ethics Committee (A1 2020/ST/105, Milan May 2020). Written informed consent was obtained from each participant before inclusion.

### 3.2. Measures

#### 3.2.1. Cognitive Assessment

All children underwent a cognitive evaluation by performing the BVN—“Neuropsychological evaluation battery for developmental age from 5 to 11 years”, an Italian standardised neuropsychological battery, which seemed to be the better choice for the current research [42]. It is structured in eight areas—language, memory, visual perception, attention, praxis, EFs, reading/writing, and calculation—each of which includes several sub-items. According to our aim, we selected ad hoc sub-items, which constituted the child registration protocol: naming on visual presentation—language area; short-term memory (digit span forward and backward)—memory area; long-term memory (free word recall)—memory area; visual attention—attention area; complex action planification and phonemic and categorical fluency—executive function area.

#### 3.2.2. Psycho-Social Investigation

Psycho-social variables were investigated through a paper-and-pen questionnaire administered to parents with the aim to gather information about their children’s demographic, psychological, emotional, and behavioural characteristics, home activities, and sleep patterns. Specifically, the questionnaire was structured as follows: (a) socio-demographic information and familiar information, (b) school history and homework, including information about the attendance of school and the autonomy in carrying out homework as well as the degree of difficulty reported in school subjects, (c) global health and COVID-19 related investigation, in which the first option was positivity of SARS-CoV-2, or if the child had never presented it, a quarantine period the child had experienced or the period of the first global epidemic wave could be selected, (d) home activities during the quarantine period, with a particular focus on sleep features, (c) psycho-motor development, including cognitive, neuropsychological, emotional-behavioural, mood, and interpersonal skills. Parents were asked to report the frequency with which the described statement occurred, using a 4-point scale (0: Never, 1: Sometimes, 2: Quite often, 3: Very often), (d) extra-curricular activities, investigating the activities that children carried out in their free time after the second COVID-19 wave between October 2020 and January 2021. This section was inserted to collect and analyse some confounding variables, for a consequent better knowledge and control.

Most of the questions were closed-ended. It was preferred to keep semi-open answers where the possibilities for answers were wide and diversified. In all sections, there was an open space for reporting any considerations that did not arise in the questions.

This survey relied on parents’ retrospective perception of feelings and behaviours their children presented before and after testing positive for SARS-CoV-2. Alternatively, they could refer to the quarantine period they experienced, or, if none of these conditions occurred, to the initial phase of the pandemic in 2020, between February and May.

#### 3.2.3. Statistics

Due to data not being normally distributed, non-parametric tests were employed to explore the associations of interest.

Wilcoxon tests were run to compare parent-reported scores evaluating children’s development before (Pre) and after (Post) the COVID-19 pandemic situation. These analyses were run both on the sample as a whole and by separately addressing participants within the CG and the NCG.

Three sets of Spearman’s correlations were run to explore the association between BVN 5–11 scores and (1) the amount of time during which children were engaged in extracurricular activities per day after the COVID-19 pandemic period and (2) parent-reported changes in children’s psycho-motor development areas, as well as between (3) the time spent on extracurricular activities and parent-reported changes in children’s psycho-motor development areas.

Data were analysed via SPSS 27.0 software (IBM Corp., Armonk, NY, USA, 2020). The significance threshold was set at α = 0.05. Missing data were excluded pairwise.

## 4. Results

Within the whole sample, a statistically significant decrease was detected only with regard to the Mood sub-section (*z* = −2.60, *p* = 0.009) (Table 2).

When separately aggressing the CG and the NCG, no pre–post differences emerged within the NCG (Table 3), whilst the same, significant decrease in Mood scores featuring the sample as a whole was likewise found in the CG (*z* = −2.35, *p* = 0.019) (Table 4). Table 5 summarises participants’ BVN 5–11 scores.

As to the association between BVN 5–11 scores and extracurricular activities, positive correlations were detected between Visual naming and Reading time (*r_s_*(22) = 0.565, *p* = 0.006), Digit span backward and Motor activity time (*r_s_*(33) = 0.45, *p* = 0.009), Visual attention and Reading time (*r_s_*(22) = 0.43, *p* = 0.048) and Phonemic fluency and Electronic device time (*r_s_*(35) = 0.37, *p* = 0.030). Moreover, a negative correlation was found between Visual attention and Graphic activity time (*r_s_*(26) = −0.42, *p* = 0.032). No other statistically significant correlations were found.

With regard to the association between BVN 5-11 scores and changes in psycho-motor development, positive correlations were detected between Digit span backward and post–pre differences in Structured behaviour (*r_s_*(40) = 0.33, *p* = 0.036) and Categorical fluency and post–pre differences in Structured behaviour (*r_s_*(40) = 0.34, *p* = 0.034). Moreover, negative correlations yielded between Visual naming and post–pre differences in Free behaviour (*r_s_*(40) = −0.32, *p* = 0.042), as well as between Free word recall and post–pre differences in Cognitive and neuropsychological skills (*r_s_*(40) = −0.43, *p* = 0.005). No other statistically significant correlations were found.

Finally, when looking at the association between changes in psycho-motor development and extracurricular activity time, positive correlations were detected between Board game time and post–pre differences in Free behaviour (*r_s_*(30) = 0.38, *p* = 0.038), as well as between Pretend play time and post–pre differences in Interaction with adults (*r_s_*(15) = 0.598, *p* = 0.019). No other statistically significant correlations were found.

## 5. Discussion

The present research directly investigated 8- and 9-year-old children’s neuropsychological abilities and emotional-behavioural abilities, with the aim to detect the presence of changes following COVID-19 pandemic consequences, such as SARS-CoV-2 infection, social isolation, and distance learning. Specifically, with regard to cognitive functioning, the main focus was on selective visual attention, short- and long-term memory, immediate verbal recall, planning, phonemics, and categorical fluency skills. The BVN 5–11 (Neuropsychological evaluation battery for developmental age from 5 to 11 years) was the evaluation tool from which the researchers selected appropriate sub-items [42]. Thanks to its administration, a first picture of the cognitive profile of the paediatric group following the COVID-19 pandemic emerged. Data collected from the whole sample of 40 children on cognitive sub-items were aligned with what has so far emerged from the literature [10,11,16], with the majority of the participants performing within or above the broad average range across most domains. A subdivision between children who were positive for SARS-CoV-2 (CG: COVID-19 group) and children who never tested positive for SARS-CoV-2 infection (NCG: non-COVID-19 group) was performed to investigate group-specific characteristics; the neuropsychological profiles of both samples were similar, with no statistically significant differences being found. In their study, Morrow and colleagues [9] found that children infected by SARS-CoV-2 show marked attentional difficulties. Through analysing this result, it should be considered that their sample was composed of children with a history of attentional problems, while in our research the children’s medical history collected through the parental questionnaire reported the absence of cognitive difficulties before the pandemic. It is possible that cognitive problems after SARS-CoV-2 infection were stronger in children with pre-existing problematic conditions, which may have worsened after infection. Furthermore, Ng and colleagues [10] in their research showed that children had difficulty in attention after SARS-CoV-2 infection, but it was not clear if the change was due to SARS-CoV-2 virus direct effects, physical symptoms such as pain, or anxiety or depression-related issues.

It is possible that, due to the absence of pre-existing problems, the difficulties of our sample were not so marked. For this reason, a cognitive investigation could be carried out using different neuropsychological batteries in addition to the BVN 5–11, in such a way to investigate attentional, memory, and executive functions and aspects in a more complete and in-depth manner [42].

The simultaneous administration of the parent questionnaire helped us to collect psychosocial information on paediatric participants. Particular attention was paid to the evaluation of the differences reported by parents in their children’s areas of development post–pre the COVID-19 pandemic. The occurrence of a negative stressful event resulted in a statistically significant change in mood of the entire sample. The CG reported a statistically significant change in mood; meanwhile, in the NCG no statistically significant differences in any of the children’s developmental areas were found.

In the aftermath of the COVID-19 outbreak, children carried out various extracurricular activities for a certain average number of minutes. From the statistical analysis emerged a predominant practice of motor activity, followed by the use of electronic devices. Similar time was spent in graphic and pretend play activities. The smallest amount of time per day was dedicated to reading activities.

Reading was positively associated with naming and attentional skills; the important role of phonological awareness, visual attention, and naming in reading development is well documented [43,44]. Moreover, children’s use of electronic devices was positively associated with verbal fluency, as reported by Arabiat et al. [45] and Capodieci and colleagues [46]. Different studies underlined that physical activity could enhance cognitive skills [47], in particular memory abilities [48]. The positive influence of motor activity on short-term memory, which emerged in the present study, fit into this theoretical framework. Furthermore, a negative correlation emerged between the time children dedicated to graphic activities and visual attention span. The use of an efficient attentional strategy has been associated with visually realistic drawings [49]. It is therefore probable that when having lower attentional skills, drawing results are less satisfactory. Children have to dedicate more time to this activity to reach an appropriate result.

The relations between neuropsychological abilities and pre–post-pandemic psychomotor development differences in children were also investigated. Particularly, children’s appropriate behaviour in structured contexts following the COVID-19 pandemic was positively correlated with short-term memory and fluency ability.

Negative correlations also emerged from the current study. A negative association between children’s behaviour in free contexts and naming ability was found; in addition, an increase in the cognitive and neuropsychological skills reported by parents was negatively correlated with long-term memory abilities as revealed by BVN 5–11 battery. However, it is important to underline that among the questions to parents, long-term memory was not specifically investigated. This result could therefore indicate a different maturation of memory, attention, and neuropsychological abilities in children. At the same time, the difference in results may be due to the discrepancy between the parents’ perception and the child’s actual functioning.

In conclusion, our results indicated a significant decrease in mood in children who tested positive for SARS-CoV-2, while no pre–post differences in children who did not experience infection were found.

Furthermore, the COVID-19 pandemic and its consequences seemed to have had no significant negative impact on the neuropsychological functioning of the examined children. In this perspective, it is possible that extracurricular activities played a protective role in children’s cognitive development.

## 6. Limitations and Future Directions

This study was conducted between 8 October and 27 November 2022. The restricted timeline of this study could have led to a small sample size, composed of self-selected subjects, and limited information, hence weakening external validity and generalisability of the study results. For future studies, extending the timeframe would help include more children/parents in more cities to better represent the targeted population. Actually, more studies are needed to better understand the impact of the COVID-19 pandemic on children’s development and mental health. In this sense, more precise knowledge of the COVID-19-related phenomena in children could be useful to develop and promote strategies aimed at preventing consequences on growth pathways.

A further limitation is that no follow-up was provided for these subjects. It would be interesting to monitor the development of the cognitive and psychosocial aspects over time to have a longitudinal overview. Additionally, the creation of a standardised questionnaire would have provided a greater psychometric quality to the survey.

Currently, no prevention or intervention programs are provided for the paediatric population. Moreover, ad hoc interventions for a small group of children with homogenous needs could be useful.

Health authorities need to adopt developmentally appropriate strategies to mitigate negative impacts on children’s well-being, including physical and mental health.

## Figures and Tables

**Table 1:** Participants’ sociodemographic characteristics (n = 40).

	No.	%
Age		
Born in 2013	13	32.5%
Born in 2014	27	67.5%
Sex		
Male	19	47.5%
Female	21	52.5%
Positivity for SARS-CoV-2		
Yes	29	72.5%
No	11	27.5%
Familiar composition		
Number of siblings		
0	9	22.5%
1	19	47.5%
2	10	25.0%
3	2	5.0%
Father		
Present	37	92.5%
Work during lockdown		
Yes	33	89.2%
Fully remote working	1	3%
Partially remote working	11	33%
In the workplace	21	64%
No	4	10.8%
Mother		
Present	38	95%
Work during lockdown		
Yes	32	84.2%
Fully remote working	5	16%
Partially remote working	9	28%
In the workplace	18	56%
No	6	15.8%

**Table 2:** Results of Wilcoxon’s test for the comparison between parent-reported scores before and after the pandemic situation.

Measure	Pre (M ± SD)	Post (M ± SD)	*z*	*p*
Cognitive and NPS	10.60 ± 2.16	10.63 ± 2.24	−0.312	0.755
Emotional skills	11.85 ± 3.46	11.58 ± 3.76	−1.41	0.158
Mood	14.98 ± 2.76	14.25 ± 3.38	**−2.60**	**0.009**
Free behaviour	10.05 ± 2.96	9.75 ± 3.01	−1.62	0.106
Structured behaviour	12.00 ± 2.54	11.93 ± 2.74	−0.535	0.593
Interaction with adults	10.83 ± 2.60	10.63 ± 2.75	−1.634	0.102
Interaction with peers	6.90 ± 1.81	6.90 ± 1.88	−0.175	0.861

Notes. Values in bold are significant. NPS = neuropsychological.

**Table 3:** Results of Wilcoxon’s test for the comparison between parent-reported scores before and after the pandemic situation within the NCG.

Measure	Pre (M ± SD)	Post (M ± SD)	*z*	*p*
Cognitive and NPS	11.45 ± 2.24	11.55 ± 2.42	−0.45	0.655
Emotional skills	12.91 ± 3.51	12.73 ± 3.41	−1.41	0.157
Mood	15.82 ± 3.57	15.36 ± 3.36	−1.13	0.258
Free behaviour	11.27 ± 2.15	11.18 ± 1.78	−0.27	0.785
Structured behaviour	12.91 ± 1.97	13.09 ± 1.76	−1.00	0.317
Interaction with adults	11.45 ± 2.38	11.27 ± 2.15	−1.00	0.317
Interaction with peers	7.36 ± 1.43	7.36 ± 1.43	0.00	1.000

Notes. NPS = neuropsychological; NCG = non-COVID-19 group.

**Table 4:** Results of Wilcoxon’s test for the comparison between parent-reported scores before and after the pandemic situation within the CG.

Measure	Pre (M ± SD)	Post (M ± SD)	*z*	*p*
Cognitive and NPS	10.28 ± 2.05	10.28 ± 2.10	−0.071	0.943
Emotional skills	11.45 ± 3.42	11.14 ± 3.84	−1.14	0.254
Mood	14.66 ± 2.38	13.83 ± 3.35	−2.35	0.019 *
Free behaviour	9.59 ± 3.12	9.21 ± 3.22	−1.68	0.094
Structured behaviour	11.66 ± 2.68	11.48 ± 2.94	−1.34	0.180
Interaction with adults	10.59 ± 2.68	10.38 ± 2.95	−1.29	0.196
Interaction with peers	6.72 ± 1.923	6.72 ± 2.02	−0.175	0.861

Notes. Values in bold are significant. NPS = neuropsychological; CG = COVID-19 group. * = statistically significant difference.

**Table 5:** Participant scores on the BVN 5–11.

	Whole Sample (M ± SD)	NCG (M ± SD)	CG (M ± SD)
Visual naming	99.34 ± 13.27	101.52 ± 10.05	98.40 ± 14.49
Digit span forward	101.68 ± 13.09	100.57 ± 15.37	102.16 ± 12.27
Digit span backward	96.79 ± 7.40	98.69 ± 6.00	95.98 ± 7.88
Free word recall	103.38 ± 13.52	101.11 ± 13.87	104.35 ± 13.50
Visual attention	101.41 ± 15.92	109.35 ± 9.09	98.01 ± 17.10
Tower of London	108.79 ± 10.34	108.07 ± 13.88	109.10 ± 8.69
Phonemic fluency	94.41 ± 13.65	93.85 ± 13.12	94.65 ± 14.10
Categorical fluency	106.22 ± 14.74	110.08 ± 12.81	104.56 ± 15.41

Notes. NCG = non-COVID-19 group; CG = COVID-19 group.

## Data Availability

Datasets related to the present study are available upon reasonable request from interested researchers.
